# Pathophysiological mechanisms underlying phenotypic differences in pulmonary radioresponse

**DOI:** 10.1038/srep36579

**Published:** 2016-11-15

**Authors:** Isabel L. Jackson, Yuji Zhang, Søren M. Bentzen, Jingping Hu, Angel Zhang, Zeljko Vujaskovic

**Affiliations:** 1 Division of Translational Radiation Sciences, Department of Radiation Oncology, University of Maryland School of Medicine, Baltimore, MD 21201, USA; 2 Division of Biostatistics and Bioinformatics, Department of Epidemiology & Public Health, and the Greenebaum Cancer Center, University of Maryland School of Medicine, Baltimore, MD 21201, USA

## Abstract

Differences in the pathogenesis of radiation-induced lung injury among murine strains offer a unique opportunity to elucidate the molecular mechanisms driving the divergence in tissue response from repair and recovery to organ failure. Here, we utilized two well-characterized murine models of radiation pneumonitis/fibrosis to compare and contrast differential gene expression in lungs 24 hours after exposure to a single dose of whole thorax lung irradiation sufficient to cause minor to major morbidity/mortality. Expression of 805 genes was altered as a general response to radiation; 42 genes were identified whose expression corresponded to the threshold for lethality. Three genes were discovered whose expression was altered within the lethal, but not the sublethal, dose range. Time-course analysis of the protein product of the most promising gene, resistin-like molecule alpha, demonstrated a significant difference in expression between radiosensitive versus radiotolerant strains, suggesting a unique role for this protein in acute lung injury.

Approximately two-thirds of cancer patients will undergo radiation therapy (RT) at some point during the course of their disease. However, the potential of RT in controlling loco-regional malignancy is limited by the risk of severe adverse effects on surrounding normal tissues. Moreover, chronic, often debilitating, treatment-related side effects often impart a significant emotional, psychosocial, and economic burden on cancer survivors, a population which counted 13,776,251 people in the U.S. on January 1, 2012^1^. Indeed, a recent report from the U.S. Centers for Disease Control found cancer survivors spend $8000 more per year on healthcare costs with productivity losses of >$3700 compared with the average population^2^. Improved understanding of the complex biological mechanisms that lead to RT-induced normal tissue injury, may in turn lead to new therapeutic strategies to prevent, mitigate, and/or treat normal tissue complications from RT.

For tumors located within the thoracic region (i.e. lung, breast, thymomas, etc.), lung damage is one of the most common normal tissue complications associated with RT due to the relatively high pulmonary sensitivity to ionizing radiation^3^,^4^. The clinical incidence of symptomatic lung injury typically varies from 5–50% depending on a number of physical, clinical, and biological parameters in particular the prescribed dose and the dose-distribution in the normal lung^5^. However, patient-to-patient variation in the development of lung injury suggest there is an underlying innate component that influences latency and severity of radiation pneumonitis/fibrosis among a genetically diverse population.

Over the past several years, we systematically characterized the pulmonary response to radiation among three genetically different, but related murine strains to select the most appropriate animal models to reflect the pathogenesis of radiation-induced lung disease (RILD) in humans and interrogate the underlying mechanisms of injury to improve target identification and test potential radiation injury mitigators for clinical translation^6^,^7^,^8^,^9^. In those studies, we defined the dose-response, latency, duration, and pathogenesis of injury in age- and sex-matched C57L/J, CBA/J, and C57BL/6J mice. Data demonstrate RILD in the C57L/J murine strain best approximated the relative dose range, time-course, and pathogenesis of injury observed in both non-human primate models and humans^8^,^9^. In contrast, the lungs of C57BL/6J (BL6) mice demonstrated a greater tolerance to radiation with a substantially longer latency period^8^,^9^.

In this study, we hypothesized that variation in pulmonary radioresponse among murine strains could be exploited to identify candidate “susceptibility” genes and/or pathways that participate in the pathogenesis of radiation injury^6^,^7^,^10^,^11^,^12^,^13^. Specifically, the objective of this study was to identify individual genes or gene patterns that represent a “switch” between tissue repair and recovery versus lethal damage using microarray technology. To this end, we performed differential gene expression analysis of lung tissue 24 hours after thoracic irradiation across the dose range where the risk of fatal radiation damage to the lung rises steeply in the two strains (C57L/J and BL6). The results identified 42 unique candidate genes and pathways which correspond to increased sensitivity to radiation damage. Next, expression of the most promising candidate gene, *Retnla*, was validated at serial time points after whole thorax lung irradiation (WTLI), covering the asymptomatic (1 day through 6 weeks) and symptomatic (to 180 days) phase in our rodent models and correlated with airway epithelial damage and inflammation.

Taken together, the results suggest tissue fate is sealed at the time of radiation exposure as a result of innate differences in cellular response to the initial insult and downstream damage. Common tissue responses, such as T-cell mediated inflammation, appear to be a consequence rather than a driver of cell damage, which can have significant implications for discovery and development of pharmacologic management strategies. Importantly, this study identified new therapeutic targets and possible biomarkers for RILD risk prediction that should be further developed in preclinical and clinical studies.

## Results

### Comparison of the radiation dose response relationship for radiation-induced lung damage in C57L/J and C57BL/6J mice

**Figure 1:**
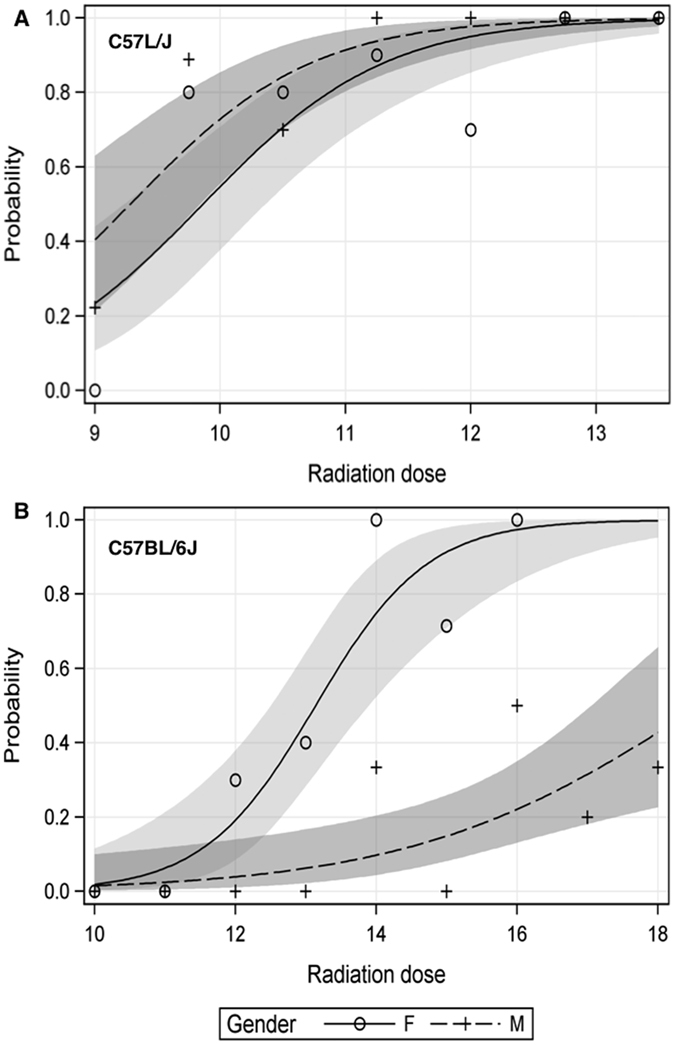
Comparison of the dose-response relationship for 180 day survival between sex-matched C57L/J and C57BL/6J mice irradiated at 10–12 weeks of age. Animals were exposed to a single graded dose of whole thorax lung irradiation (320 kVp x-rays, 1.25 cGy min^−1^, HVL = 1 mm Cu) and observed for signs of major morbidity/mortality over the first 180 days post-exposure. The dose-response relationship demonstrates a lower threshold (~9.75 Gy females vs. 9.0 Gy, males) for lethal radiation pneumonitis by day 180 in C57L/J mice (**A**) compared to C57BL/6J mice (**B**)(~12.0 Gy, females vs. 14 Gy, males). In both strains the dose-response is steep with 100% lethality at 12.75 and 11.25 Gy in female and male C57L/J mice, respectively, and 16 Gy in female C57BL/6J mice. In C57BL/6J mice, the lethal dose for 100% of males within the first 180 days is >17Gy.

In C57L/J mice irradiated at 10–12 weeks of age, the incidence of lethal pneumonitis/fibrosis increases sharply over a range of 9.0 to 12.0 Gy WTLI, with no significant difference observed between female versus male mice in 180-day survival (p = 0.13, Logistic Regression) (Fig. 1A). In contrast, sex has a dose-modifying effect on 180-day morbidity/mortality in C57BL/6J mice irradiated at 10–12 weeks of age. For every 1 Gy increase in radiation dose, the odds of dying for female mice by day 180 is 3.54 (p < 0.0001) and 1.62 in male mice (p = 0.003, Logistic Regression) compared with unirradiated animals. The threshold dose for major morbidity/mortality after WTLI in C57BL/6J mice is approximately 12 Gy in females vs. 14 Gy in males (Fig. 1B).

### Differential gene expression analysis identified 805 genes commonly expressed as a general response to radiation in C57L/J and C57BL/6J mice

**Figure 2:**
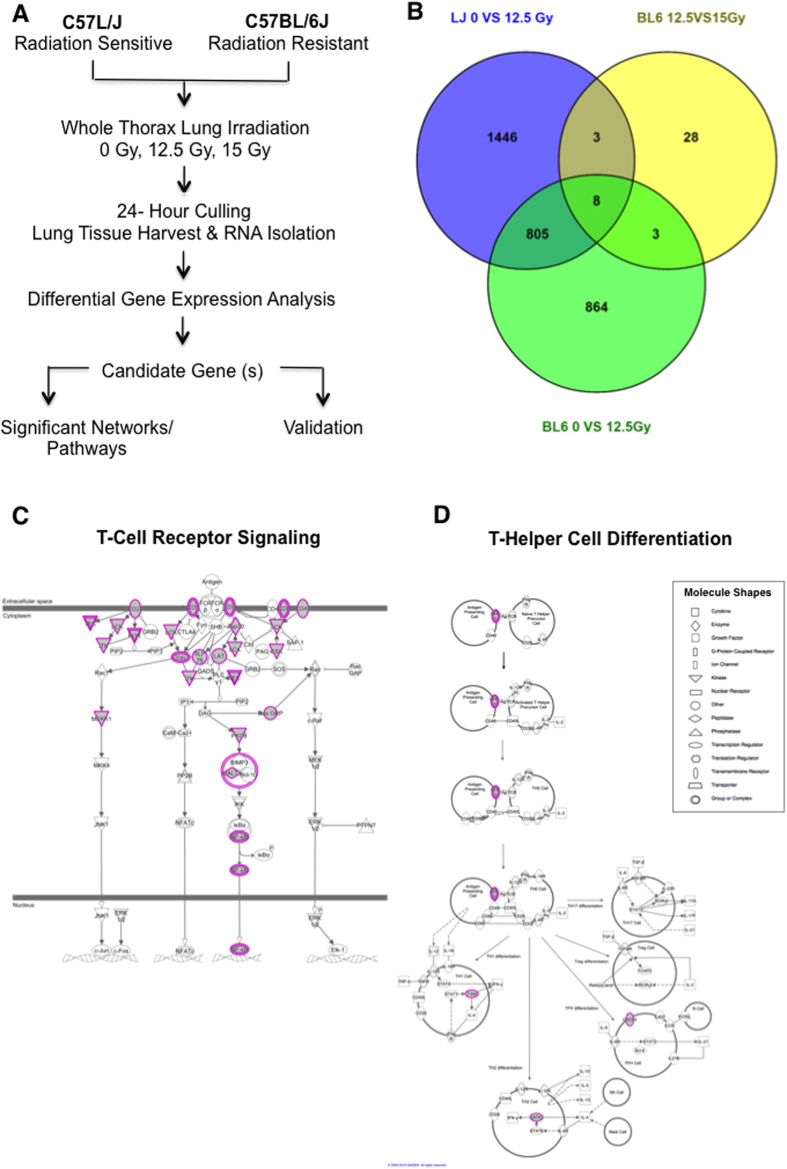
Differential gene expression profile of pulmonary radioresponse by murine strain and radiation dose. (**A**) Age-matched (10–12 week old) female C57L/J and C57BL/6J mice were irradiated to the whole thorax with a single dose of 12.5 or 15 Gy of 320 kVp X-rays (67 cGy min^−1^, 1 mm Cu HVL). Sham-irradiated (0 Gy) animals were included as controls. Twenty-four (24) hours post-irradiation, lung tissue was harvested and RNA isolated from the right upper lobe. For differential gene expression analysis, mouse oligonucleotide arrays were printed at the Duke Microarray Core Facility using Affymetrix Mouse Genome 430 2.0 gene chips. Samples (n = 3 per strain per dose) were not pooled rather run independently for a total of 27 arrays. Raw microarray profiling data was processed, normalized, and analyzed as described in the Methods section. The criteria for inclusion of an mRNA in the differentially expressed gene list was set to p-value < 0.01. The analysis was performed using BRB-ArrayTools. Differentially expressed mRNA lists were annotated using QIAGEN’s Ingenuity^®^ Pathway Analysis. (**B**) Venn Diagram showing the overlap of genes commonly and differentially expressed among murine strains post- 0, 12.5, or 15 Gy WTLI. Three genes, *Retnla, Gria1,* and *Auts2,* were found to be commonly expressed across the dose range for lethal lung injury in both strains. (**C**) T-cell receptor signaling pathway and (**D**) T-helper cell differentiation were two of the top enriched networks altered in response to radiation in both strains. Genes within each pathway whose expression is altered in response to radiation are highlighted in purple. The networks were generated through the use of Qiagen’s Ingenuity Pathway Analysis (IPA^®^, Qiagen Redwood City, www.qiagen.com/ingenuity).

Due to the shift in the position of the dose-response curve, we designated C57L/J mice as “radiation sensitive” and C57BL/6J mice as “radiation resistant” for the purpose of comparison. To elucidate differential gene expression patterns in sham versus irradiated lungs across the dose-range that results in lethality in C57L/J and/or C57BL/6J mice, we exposed mice to a single dose of 0 Gy, 12.5 Gy, or 15 Gy of 320 kVp x-rays to the whole thorax (dose rate 67 cGy min^−1^, HVL = 1 mm Cu). Animals were culled twenty-four hours after exposure for differential gene expression analysis (n = 3/dose/strain). Messenger RNA (mRNA) was extracted and global changes in the transcriptome profile examined using Affymetrix oligo arrays to identify candidate genes and/or pathways associated with development of RILD (Fig. 2A).

DNA microarray expression profiling identified 805 genes altered in response to radiation in both C57L/J and C57BL/6J (BL6) mice (Fig. 2B). There were a relatively limited number of 42 genes which showed a statistically significant change in expression in the critical dose range from 12.5 to 15 Gy in the BL6 strain (Fig. 2B). Hierarchical cluster analysis revealed distinct patterns of gene expression in C57L/J and C57BL/6J mice reflecting a common signature for pulmonary radioresponse (Supplemental Material, Fig. 1). Some of the radioresponsive genes identified are fundamental to the response to ionizing radiation in mammalian cells, for example Cdkn1a, a gene tightly controlled by p53 that encodes a protein mediating cell cycle arrest via the G1/S and G2/M check points (Table 1). Genes significantly differentially expressed between sham-irradiated versus irradiated lungs of both strains enriched in pathways and interaction networks related to T-cell mediated immune response, including T-cell receptor signaling (Fig. 2C) and T-helper cell differentiation (Fig. 2D).

### Dose and strain dependent changes in gene expression identify three genes significantly differentially expressed across the dose range for lethality in both C57L/J and C57BL/6J mice

**Figure 3:**
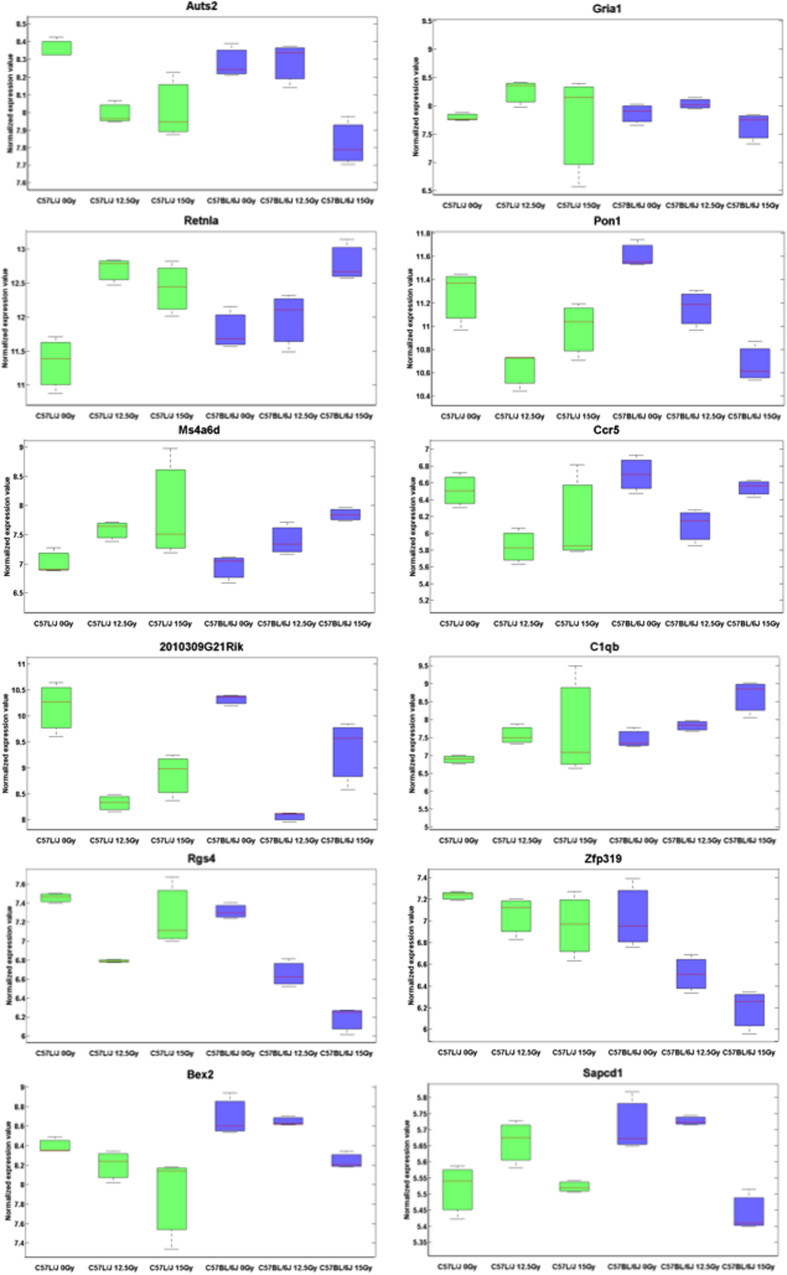
Relative mRNA expression of selected genes differentially expressed in response to radiation by murine strain and radiation dose. Box plot of genes downregulated (*Auts2, Gria1)* or upregulated *(Retnla)* across the dose-range for lethal radiation pneumonitis in both C57L/J and C57BL/6J mice. Other genes exhibited a radiation dose- and/or strain dependent change in expression in murine lungs. The full list of 42 genes is provided in Tables 2 and 3. The y-axis represents Log2(expression).

To identify genes with altered expression specifically across the critical dose range for major morbidity/mortality over the first 180 days postWTLI, we compared gene expression patterns between C57L/J and BL6 mice at 0 vs. 12.5 Gy and 12.5 Gy vs 15 Gy in BL6 mice. These three genes-*Auts2* (P value < 0.008, 0.003 respectively)*, Gria1* (P value < 0.005, 0.007 respectively), *and Retnla* (P value < 9.39E-05, 0.009 respectively)*–* not previously identified in the pathobiology of normal tissue response to radiation–were differentially expressed in the lungs of both C57L/J and BL6 mice in the lethal dose range (0 vs. 12.5 Gy and 12.5 Gy vs. 15 Gy respectively), but not in the sublethal dose range (0 vs. 12.5 Gy in BL6 mice) (Fig. 3). Here, loss of Auts2 expression and increased *Retnla* expression corresponded specifically to the threshold dose for lethality from RILD over the first 180 days in both strains. Gria1 was less conclusive, with the mean fold change in gene expression suggesting an increase in expression in C57L/J mice (12.5 and 15 Gy); and a decrease in expression in BL6 mice (15 Gy). Eight genes demonstrated radiation dose-dependent changes in gene expression in the lungs of both C57L/J and BL6 mice. Three genes–*Specc1, Zfp319, Cyp26b1*–were found to exhibit a dose-dependent change in expression at p < 0.01 in the lungs of BL6 mice only (Table 2). Gene ontology annotation for biological process and KEGG pathway analysis of genes exhibiting a dose-dependent change in gene expression is shown in Table 2. A total of 28 genes were shown differently expressed in the lungs of BL6 mice at 15 Gy, but not 12.5 Gy using the cut-off criteria of both p < 0.01 and >2-fold change in expression (Table 3).

### Resistin-like molecule alpha expression (RELM-α in lung tissue after thoracic irradiation

**Figure 4:**
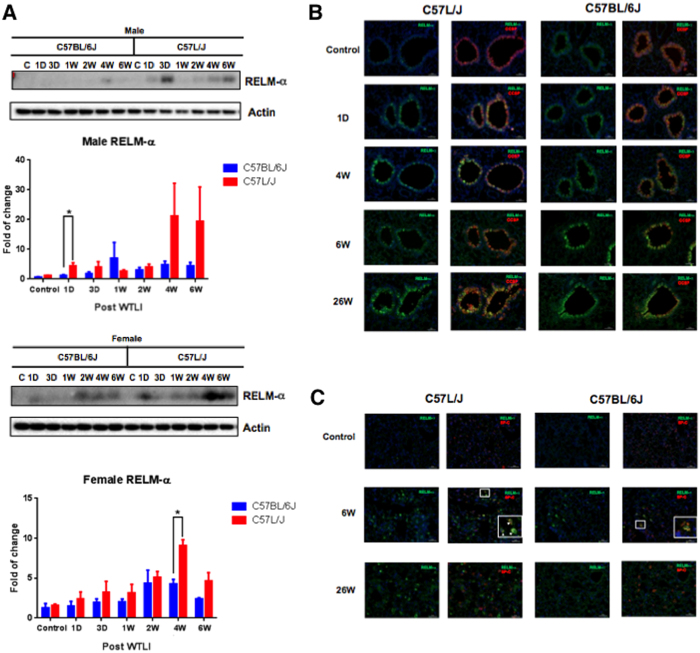
Time and sex-dependent changes in Resistin Like Molecule –Alpha (RELM-α) expression in irradiated lungs of C57L/J and C57BL/6J mice. (**A**) Quantitative analysis of RELM-α protein expression in the lungs of male and female C57BL/6J and C57L/J mice following a single dose of 15 Gy whole thorax lung irradiation based on western blot data. Values represent mean ± SEM. Expression of RELM-α co-localized with club (formerly known as clara) cells indicated by CCSP staining (**B**), but not alveolar epithelial type 2 (AEC2) cells as indicated by SP-C staining (**C**).

Among the three genes differentially expressed at the dose-response threshold for lethal RILD in both strains of mice, *Rtnla* is of particular interest. This gene encodes for the protein resistin-like alpha (RELM-α) also known as found in inflammatory zone 1 (FIZZ1) and hypoxia-inducible mitogenic factor (HIMF). A separate cohort of age- and sex-matched C57L/J and C57BL/6J mice (10–12 weeks of age at the time of irradiation) were exposed to 0 or 15 Gy WTLI (320 kVp x-rays, 1.25 Gy min^−1^, HVL = 1 mm Cu). Animals were culled at 1 and 3 days, 1, 2, 4, and 6 weeks, and 26 weeks post-exposure. Serial analysis of RELM-α protein expression over time demonstrated a similar increase in RELM-α in the lungs of female C57L/J mice as early as 24 hours after a single dose of 15 Gy WTLI with a surge in expression at 4 and 6 weeks post-exposure (Fig. 4A). Consistent with the microarray data, an increase in RELM-α was observed in the lungs of female BL6 mice exposed to 15 Gy. A similar increase was not observed in the lungs of male BL6 mice following a dose of 15 Gy WTLI, which is below the threshold for lethal incidence of RILD in this strain (LD50/180 = 13.1 Gy vs. 18.8 Gy, female vs. male, respectively). Further analysis demonstrated RELM-α co-localized with bronchiole epithelial damage, specifically club cells (Fig. 4B). At 180 days, RELM-α co-localized with bronchiole epithelial cells and inflammatory cells, but not ATII cells (Fig. 4C).

### Expression of RELM-alpha, in lung tissue 180 days post-exposure to 15 Gy WTLI

**Figure 5:**
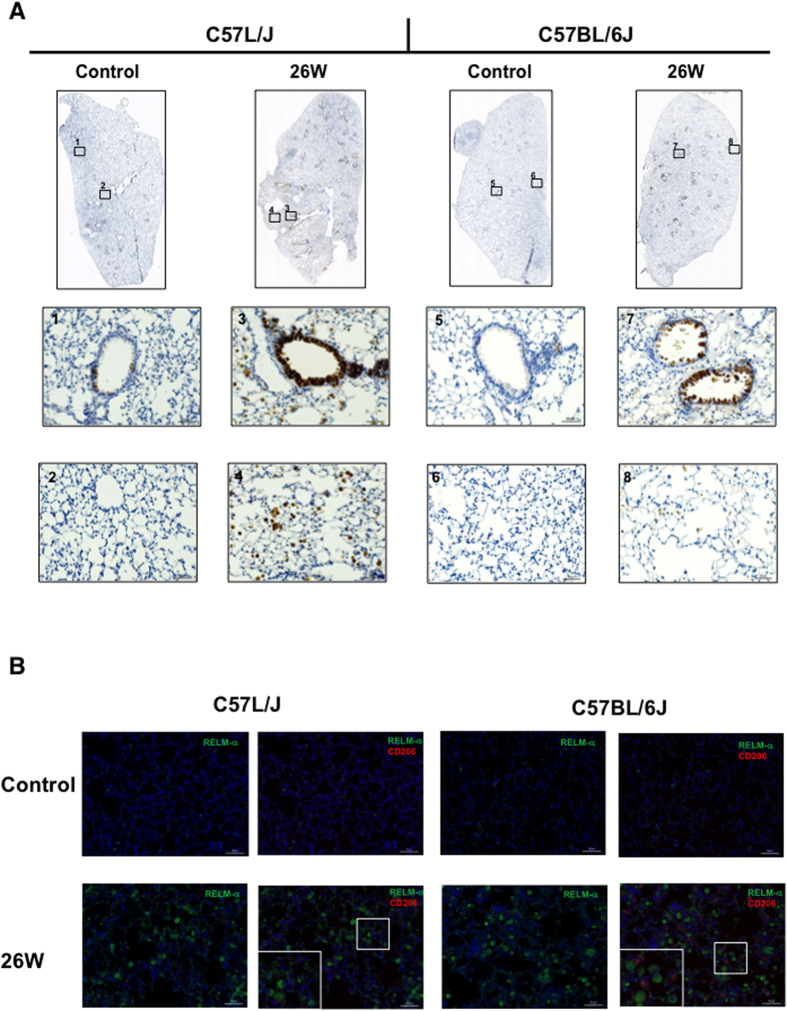
Expression of Resistin Like Molecule –Alpha (RELM-α), a marker of alternatively activated macrophages, during the fibrosis phase of radiation-induced lung injury. (**A**) RELM-α expression in the lungs of sham-irradiated (control) or irradiated C57L/J and C57BL/6J mice at 26 weeks post-exposure. Abundant expression is observed in the bronchioles and airways of irradiated, but not sham-irradiated lungs. The intensity of expression was greater in C57L/J mice than in C57BL/6J mice. (**B**) CD206, also known as mannose receptor type 1, is a marker of alternatively activated macrophages. A high number of alternatively-activated macrophages are observed in the fibrosis-prone lungs of C57BL/6J mice, however, most of them did not express RELM-α. Very few alternatively activated macrophages were observed in the lungs of C57L/J mice, who develop a less robust fibrotic reaction in response to radiation.

Immunostaining demonstrated a difference in RELM-α expression between the lungs of sham-irradiated and irradiated (15 Gy) C57L/J and to a lesser extent C57BL/6J mice (Fig. 5A). Positive staining (brown) was observed around the large airways and resident macrophages. In the evaluated tissue sections, RELM-α a well-known marker of alternatively activated macrophages, RELM-α did not co-localize with macrophage infiltration at 26 weeks (Fig. 5B).

## Discussion

In this study, experiments were designed to compare and contrast gene expression in sham-irradiated and irradiated lungs of C57L/J and C57BL/6J mice twenty-four hours after whole thorax lung radiation (WTLI). These murine strains were chosen based on their well-characterized dose-response and phenotypic expression of RILD^6^,^8^,^9^. For the purposes of this study, we define the lungs of the C57L/J strain as radiosensitive and the lungs of C57BL/6J mice as radiotolerant due to the separation in the radiation dose-response and pathogenesis of injury between the two strains (Fig. 1). We hypothesized gene expression profiling of radiosensitive vs. radiotolerant lungs would identify actionable targets for therapeutic intervention and/or candidate biomarkers to predict individual risk for development of RILD.

Genome-wide transcriptome analysis revealed a number of common genes (n = 805) differentially expressed in response to radiation across both strains (Fig. 2A and Supplemental Material, Fig. 1). Pathway enrichment analysis of commonly expressed genes pointed to the importance of DNA damage/repair/cell death and T-cell mediated immune responses in the acute normal tissue response to irradiation (Table 1, Fig. 2C,D). That T-cells play a major role in RILD is well-established^14^,^15^,^16^. In preclinical studies, the severity of pneumonitis has been shown to be significantly reduced in C57BL/6J mice exposed to total body irradiation with bone marrow transplant after thymectomy^17^. However, despite these data, the actual role of T-cell mediated inflammatory responses in inherent radiosensitivity and divergent outcomes in the pathogenesis of RILD is unclear. The commonality of the response between sensitive and resistant strains suggests targeting T-cell mediated immune response may modify the symptoms acutely, but is unlikely to mitigate the underlying pathogenesis. This is supported by the ineffectual treatment of RILD with corticosteroids clinically and other anti-inflammatories preclinically.

Deeper analysis of differential gene expression over the dose-response range that results in sublethal vs. lethal pneumonitis/fibrosis, identified only three genes associated with the threshold for lethality in both strains- *Retnla*, *Gria1*, and *Auts2* (Fig. 3). Unlike *Gria1* and *Auts2* where little is known about their role in inflammation and pulmonary disease, the protein product of *Retnla* has been shown to be upregulated by chronic hypoxia in lung tissue, a hallmark feature of RILD^18^,^19^,^20^, through an IL-4 dependent mechanism^21^,^22^. As such, it was selected for further analysis in this study.

The *Retnla* gene encodes for resistin-like molecule alpha (RELM-α/FIZZ1/HIMF) 9.4 kDA cysteine-rich secreted protein that has been shown to induce endothelial cell apoptosis^23^, facilitate airway and vascular remodeling^21^,^24^, promote inflammation^22^, and modulate myofibroblast differentiation in allergic pulmonary inflammation and fibrotic lung disease^25^.

Serial analysis of tissue collected in separate experiments confirmed the significant increase in RELM-α expression in the irradiated lungs of female C57L/J and C57BL/6J mice at 24 h post WTLI, but not in the more radiotolerant lungs of male C57BL/6J (Fig. 4A). This is consistent with the lack of a difference in radiation dose-response between the lungs of female versus male C57L/J mice, whereas there is a significant sex-difference observed in C57BL/6J mice (p < 0.0001) (Fig. 1B). Dual fluorescent immunostaining demonstrated a time-dependent increase in RELM-α expression within the airways of irradiated lungs of both C57L/J and C57BL/6J mice (Fig. 4B). Expression was strongly co-localized with club cells in C57L/J mice and to a lesser extent in BL6 mice (Fig. 4B). Type II alveolar epithelial cells in the irradiated lungs of 50% (n = 2/4) of C57L/J and 25% (n = 1/4) of BL6 mice expressed RELM-α during the symptomatically latent period of disease progression (6 weeks) but not at 26 weeks postWTLI (Fig. 4C). In this study, alternatively activated macrophages (AAM) were observed in the lungs of fibrosis-prone C57BL/6J mice, but not C57L/J mice. Although RELM-α was abundantly expressed in the airways of irradiated lungs, expression was not observed to be co-localized with alternatively-activated macrophages at the time of pneumonitis/fibrosis (Fig. 5). This was an unexpected finding as RELM-α is often utilized as a marker of alternatively activated- macrophages, which are known to promote collagen deposition and fibrosis^25^. This finding may in part be a result of the rapid secretion of RELM-α^26^. In other studies, a significant correlation between macrophage infiltration/alveolar inflammation and fibrosis has been observed suggesting alternatively-activated macrophages may play a significant role in fibrosis^8^. Taken together, it is tempting to hypothesize that C57BL/6J mice exhibit greater wound repair and anti-inflammatory activity than C57L/J mice.

We hypothesize the early increase in RELM-α expression is a result of an acute onset of tissue hypoxia due to significant morphological abnormalities observed in mouse lungs twenty-four hours post-WTLI. Electron micrographs show lethal cell injury, interstitial cell necrosis, Type II cell apoptosis, bronchial epithelial denudation, disruption of the basement membranes, and endothelial cell swelling within the irradiated lungs of female C57L/J mice that result in vascular occlusion and impaired gas exchange. In contrast, ultrastructural changes were less severe in C57BL/6J mice and in neither strain were changes evident histologically. This is consistent with our previous findings showing tissue hypoxia develops within the first few days post-WTLI^18^,^19^.

Whether RELM-α upregulation is causative of lethal RILD or whether it is simply a biomarker for passing the “point of no-return” due to the severity of tissue damage is unclear. We hypothesize acute tissue hypoxia due to cell injury/death and later development of chronic hypoxia due to lymphocytic alveolitis and fibroproliferation within the irradiated lung tissue may promote RELM-α expression. These data, coupled with recent data collected in our lab on cell kinetics postWTLI, suggest differences in inherent radiosensitivity do not appear to be the result of different target cell populations between the various strains, but are related to differences in innate cellular radiosensitivity as well as the speed and integrity of epithelial repair. Future studies will seek to elucidate the specific role of RELM-α in the pathogenesis of normal tissue damage following radiation exposure and determine whether targeting RELM-α can mitigate the severity of RILD.

In summary, comparing and contrasting differences in radiation-induced gene expression between strains identified actionable targets that were previously unrecognized as playing a role in pulmonary effects of radiation exposure. Further studies will explore the role of selected genes and their protein products in modifying lung damage–either towards increased radiosensitivity or increased radiotolerance among various murine strains–with the aim of identifying new treatments for RILD.

## Methods

### Animals and Radiation Exposure

For differential gene expression analysis, female C57BL/6J and C57L/J mice (Jackson Labs, Bar Harbor, ME) were irradiated at 10–12 weeks of age with 12.5 or 15 Gy of 320-kVp X rays (Precision X-ray Inc., North Branford, CT, HVL = 1.00 mm Cu dose rate = 67 cGy min^−1^). Age-matched sham-irradiated mice were included as controls. Unexposed regions were shielded using 8-mm lead shielding. Mice were euthanized twenty-four hours post-exposure by pentobarbital overdose (>250 mg/kg). Lung tissue was excised, embedded in OCT, and frozen over dry ice. Tissue was stored at −80 °C until analysis.

For serial tissue analysis, age and sex-matched C57BL/6J and C57L/J mice at 10–12 weeks of age were irradiated to the whole lung with a single dose of 15 Gy of 320 kVp X-rays at a dose rate of 1.25 cGy min^−1^ (Precision X-ray Inc., North Branford, CT, HVL = 1.00 mm Cu). Sham-irradiated age and sex matched animals were included as controls. Animals (n = 10/group) were culled at 1 day, 3 days, 1 week, 2 weeks, 4 weeks, 6 weeks, and 26 weeks post-exposure. All animal experiments were conducted with prior approval from the IACUC at the Duke University and University of Maryland School of Medicine.

All procedures involving the mice were performance in accordance with the animal protocols approved by the Institutional Animal Care and Use Committees (IACUC) at the Duke University Medical Center and the University of Maryland School of Medicine and complied with all institutional, state, and federal regulations. The maintenance and surveillance of animals involved a high standard of humane care at a level above recommendations set forth in the NIH publication “Guide for the Care and Use of Laboratory Animals”. The method of euthanasia at both institutions was consistent with the recommendations of the Panel on Euthanasia of the American Veterinary Medical Association^27^ and in accordance with IACUC requirements at both institutions.

### RNA Isolation

RNA isolation was performed using the Qiagen RNeasy kit (Qiagen, Valencia, CA) according to the manufacturer’s protocol with slight modifications^28^. At the time of RNA extraction, the right upper lobe from three to four mice per group was excised from OCT, placed in RNALater for five minutes, and homogenized in 2 mL of lysis buffer (Qiagen, Valencia, CA) with zirconia-silica beads using a BeadBeater (BioSpec., Bartlesville, OK). The samples were not pooled. RNA quality was assessed using the Agilent Bioanalyzer 2100 (Agilent Technologies, Palo Alto, CA) located at the Duke Microarray Facility. Only RNA with an RNA Integrity Number (RIN) above 9.0 was used.

### Affymetrix mouse gene chip hybridization

Mouse oligonucleotide arrays (1 array per RNA sample for a total of 27 arrays) were printed at the Duke Microarray Core Facility using Affymetrix Mouse Genome 430 2.0 gene chips, which allows 39,000 transcripts to be analyzed in a single array. All GeneChip Mouse Genome Arrays from Affymetrix contain a set of mouse maintenance genes to facilitate normalization of array experiments prior to performing data comparisons. Amplification, probe preparation and hybridization protocols were performed using the MessageAmp**™** Premier RNA Amplification kit (Applied Biosystems, Foster City, CA)^29^.

### Data Normalization and Quality Control

To guard against batch effects or other technical factors impacting array data, we took the following precautions. Animals were maintained under identical housing conditions and euthanized on the same day. Mice were irradiated in groups of 10 and alternated by strain along the radiation platform to minimize effects due to non-uniform radiation distribution or internal errors in the radiation procedure. Previous radiation field uniformity tests indicate less than 6% difference across the field. Samples were processed and hybridized in a single batch to protect against batch effects. RNA extraction and preprocessing methods used in this study are well characterized. To ensure reproducibility and minimize error, samples (n = 3 per radiation dose for each strain) were not pooled, rather run independently.

### Data Analysis

Raw microarray profiling data was preprocessed and quartile-normalized. To identify differentially expressed mRNAs between groups, a random variance t test was used aiming to improve estimates of gene-specific variances without assuming that all genes have the same variance^30^. The criteria for inclusion of an mRNA in the differentially expressed gene list was set to p-value < 0.01. The analysis described was performed by using BRB-ArrayTools Version 4.4.0 developed by Dr. Richard Simon and the BRB-ArrayTools Development Team.

The differentially expressed mRNA lists were further annotated through the use of QIAGEN’s Ingenuity^®^ Pathway Analysis Version 26127183 (IPA^®^, QIAGEN Redwood City, www.qiagen.com/ingenuity). We queried IPA with the differentially expressed mRNA list aiming to map and generate putative biological processes/functions, networks and pathways based on the manually curated knowledge database of molecular interactions extracted from the published literature. The differentially expressed genes were overlaid onto a global molecular network developed from direct/indirect interaction information documented in the Ingenuity Knowledge Base. Based on a right-tailored Fisher’s exact test, the functional analysis identified canonical pathways, biological functions and/or diseases that were most significant to the data set (p < 0.05). These pathways and networks were then ranked by the enrichment score that measures the probability that the genes were included in the network by chance.

### Immunoflourescent and immunohistochemical staining

The left mouse lung was perfused, fixed with 10% PFA and embedded in paraffin. The tissue sections were subjected to deparaffinization, dehydration and antigen retrieval in citrate buffer at 95 °C. After blocking with 10% normal donkey serum, 1% BSA and 5 µg/ml mouse BD Fc block^TM^ in PBS, the sections were incubated with rabbit anti-murine RELM-alpha (#500-P214, PeproTech, 1/2000), goat anti-clara cell protein (sc-9772, Santa Cruz Biotechnology, 1/400), goat anti-SP-C (sc-7706, Santa Cruz Biotechnology) and Rat anti-mouse CD206 : Biotin (MCA2235BT, AbD Serotec, 1/50). The Alexa Fluor 488-conjugated donkey anti-rabbit antibody (A21206, Life Technologies, 1/500), Alexa Fluor 647-conjugated donkey anti-goat antibody (A21447, Life technologies, 1/500), and streptavidin Alexa Fluor 647 conjugate (S32357, Life technologies, 1/200) were used to visualize the antigenic signal. For immunohistochemistry (IC) staining, the antigenic signal was visualized by the incubation with ImmPRESS anti-rabbit IgG HRP (MP-7401) as secondary antibody followed by a reaction with ImmPACT DAB peroxidase substrate (SK-4105, Vector Laboratories).

### Western Blotting

The mouse lung tissues were homogenized in ice-cold RIPA buffer (Cell Signaling) containing complete protease inhibitor cocktail (Roche Diagnostics GmbH) using TissueLyser LT (QIAGEN). The protein concentration in the lysate was determined using the Pierce^TM^ BCA Protein Assay Kit (Thermo Scientific). After being heated at 70 °C for 10 min in a tricine sample buffer with β-Mercaptoethanol, the proteins were separated by Criterion^TM^ tris-tricine/peptide gel electrophoresis. And then, the proteins were transferred to PVDF membrane by Trans-Blot Turbo^TM^ Blotting system (Bio-Rad). The membranes were incubated with primary antibodies, such as a rabbit anti-murine RELM-α (500-P214, PeproTech, 1/400) or goat β-actin polyclonal antibody (sc-1616, Santa Cruz Biotechnology, 1/1000) at 4 °C overnight. After washing, the membranes were incubated with anti-rabbit (#7074, Cell Signaling, 1/1000) or anti-goat (sc-2350, Santa Cruz Biotechnology, 1/5000) HRP-conjugated secondary antibodies. The signal was visualized by SuperSignal^®^ West Femto Maximum Sensitivity Substrate (Thermo Scientific). The images were quantified using ImageLab software (Bio-Rad). Results are presented as mean ± SEM. The difference between the two strains C57BL/6J and C57L/J was analyzed by student t-test. P < 0.05 was considered significant.

### Data availability

Raw microarray expression files are publicly available from the Gene Expression Omnibus (GEO) database. The GEO Accession Number is GSE85359.

## Additional Information

**How to cite this article**: Jackson, I. L. *et al.* Pathophysiological mechanisms underlying phenotypic differences in pulmonary radioresponse. *Sci. Rep.* **6**, 36579; doi: 10.1038/srep36579 (2016).

**Publisher’s note**: Springer Nature remains neutral with regard to jurisdictional claims in published maps and institutional affiliations.

## Supplementary Material

Supplementary Information

## Figures and Tables

**Table 1:** Top ingenuity canonical pathways enriched by genes that were significantly differentially expressed in the lungs of both murine strains in response to radiation.

Canonical Pathway	P-Value	Ratio^1^	Molecules
CD28 signaling in T helper cells	7.943282E-19	2.63E-01	CD247, HLA-DOA, PIK3R5, HLA-DQA1, HLA-DQB1, NFKB1, PTPRC, CD28, LCK, HLA-DMA, PIK3CG, HLADMB, ITK, PTPN6, PRKCQ, ITPR2, CSK, MAP3K1, MALT1, CD3D, CD3G, WAS, SYK, ZAP70, LAT, HLA-DOB, CD86, VAV1, PIK3CD, LCP2, HLA-DRB5
T cell receptor signaling	3.162278E-14	2.47E-01	CD247, PRKCQ, CSK, MAP3K1, PIK3R5, MALT1, NFKB1, CD8A, CD8B, CD3D, TEC, BTK, PTPRC, CD3G, CD28, LCK, PIK3CG, RASGRP1, LAT, ZAP70, VAV1, PIK3CD, LCP2, ITK
Role of NFAT in regulation of the immune response	5.011872E-14	1.81E-01	CD247, BLNK, HLA-DOA, PIK3R5, HLA-DQA1, HLA-DQB1, NFKB1, CD28, LCK, HLA-DMA, PIK3CG, HLA-DMB, ITK, PRKCQ, CD79B, ITPR2, CD79A, CD3D, BTK, CD3G, SYK, PLCG2, LAT, ZAP70, HLA-DOB, CD86, MEF2C, PIK3CD, GNAL, LCP2, HLA-DRB5
B-cell development	3.162278E-12	4.12E-01	CD19, HLA-DOA, SPN, CD79B, HLA-DQA1, CD79A, HLA-DQB1, IL7R, PTPRC, HLA-DMA, HLA-DMB, HLA-DOB, CD86, HLA-DRB5
T helper cell differentiation	3.162278E-11	2.54E-01	HLA-DOA, IL21R, HLA-DQA1, HLA-DQB1, TBX21, STAT4, CD28, HLA-DMA, TGFB1, HLA-DMB, ICOS, IL10RA, HLA-DOB, CD86, CXCR5, GATA3, ICOSLG/LOC102723996, HLA-DRB5
CTLA4 signaling in cytotoxic T lymphocytes	1.412538E-09	2.05E-01	HLA-DOA, SLAMF1, CD79B, HLA-DQA1, LTB, CD79A, HLA-DQB1, NFKB1, TNFRSF13C, CD28, HLA-DMA, TGFB1, TLR1, HLA-DMB, HLA-DOB, CD86, TNFRSF13B, HLA-DRB5
p53 signaling	3.981072E-05	1.33E-01	TP53INP1, GADD45G, PIK3R5, MDM2, BAX, ST13, BIRC5, SERPINE2, CCNG1, BBC3, PIK3CG, CDKN1A, PIK3CD
Thrombin signaling	1.513561E-04	9.42E-02	PRKCQ, CAMK1D, ITPR2, PDIA3, ARHGEF15, PIK3R, NFKB1, MAPK11, RHOH, PLCG2, PIK3CG, PIK3CD, GATA3, PLCD4, FNBP1, GNAL, PRKCA, PRKCB
Role of Pattern Recognition Receptors in Recognition of Bacteria and Viruses	1.584893E-04	1.1E-01	PRKCQ, PIK3R5, C1QA, CCL5, NFKB1, TGFB1, PLCG2, PIK3CG, SYK, TLR1, PIK3CD, EIF2AK2, PRKCB, PRKCA
Cell cycle: G2/M DNA damage checkpoint regulation	2.884032E-04	1.63E01	CDC25B, CDKN1A, TOP2A, CCNB2, MDM2, PLK1, RPS6KA1, CDK1
Protein kinase A signaling	6.165950E-04	6.99E-02	HIST1H1C, PDIA3, DUSP6, UBASH3B, NFKB1, DUSP2, PTPRC, CDC25B, TGFB1, PTPRO, PLCD4, PRKCA, PTPN6, PRKCQ, ITPR2, MAP3K1, PTCH1, PTPN18, PTPDC1, AKAP13, ADD3, PLCG2, CREM, LEF1, Tcf7, PTPN22, PRKCB
NF-κB Signaling	1.258925E-03	8.67E-02	PRKCQ, MAP3K1, PIK3R5, MALT1, NFKB1, IGF2R, LCK, PLCG2, PIK3CG, TLR1, ZAP70, PIK3CD, TRAF5, EIF2AK2, PRKCB
Sphingosine-1-phosphate signaling	1.621810E-03	1.01E-01	S1PR4, PDIA3, PLCG2, PIK3CG, CASP2, SPHK1, PIK3R5, PIK3CD, RHOH, PLCD4, FNBP1

^1^The ratio indicates the total number of differentially expressed genes in our data set that overlap with the molecules in the canonical pathway over the total number of all genes annotated in that same canonical pathway.

**Table 2:** Genes showing dose-dependent changes in expression in the lungs of C57BL/6J mice following 12.5 or 15 Gy WTLI.

Gene Symbol	Gene Name	Fold-Change 0 vs. 12.5 Gy	P-Value	Gene Ontology Biological Process
Specc1	sperm antigen with calponin homology and coiled-coil domains 1	1.56	p = 0.002	Unknown
Zfp319	zinc finger protein 319	1.51	p = 0.006	regulation of transcription, DNA-templated
Cyp26b1	cytochrome P450, family 26, subfamily b, polypeptide 1	3.00	p = 0.001	Regulation of T-cell differentiation; Inflammatory response
Rgs4	regulator of G-protein signaling 4	1.58	p = 0.0002	G-protein coupled receptor signaling pathway
Pon1	paraoxonase 1	1.37	p = 0.004	Aromatic compound catabolic process
Ms4a6d	membrane-spanning 4-domains, subfamily A, member 6D	0.71	p = 0.005	Unknown
Ccr5	chemokine (C-C motif) receptor 5	1.53	p = 0.003	Positive regulation of IL-1beta secretion; immune response
Ighm	immunoglobulin heavy constant mu	2.77	p = 0.003	B cell receptor signaling pathway
2010309G21Rik	Uncharacterized protein	4.73	p < 1e-07	Unknown
C1qb	complement component 1, q subcomponent, beta polypeptide	0.72	p = 0.006	Complement activation, classical pathway
Batf3	basic leucine zipper transcription factor, ATF-like 3	1.58	p = 6.41 e-05	Dendritic cell differentiation

**Table 3:** Genes differentially expressed in the lungs of C57BL/6J mice over the lethal IR dose range (12.5–15 Gy), but not sublethal (0–12.5 Gy) range.

Symbol	Name	Fold Change 12.5 vs. 15 Gy	P-Value 12.5 vs. 15 Gy	Gene Ontology Biological Process/Function
Bex2	Brain expressed X-linked 2	1.32	p = 0.002	Regulator of mitochondrial apoptosis and G1 cell cycle in breast cancer
Slc16a6	Solute carrier family 16 (monocarboxylic acid transporters), member 6	0.77	p = 0.009	Proton-linked monocarboxylate transporter
Arhgef28	Rho guanine nucleotide exchange factor (GEF) 28	1.27	p = 0.010	Functions as a RHOA-specific guanine nucleotide exchange factor regulating signaling pathways downstream of integrins and growth factor receptors.
Tnfrsf17	Tumor necrosis factor receptor superfamily, member 17	0.76	p = 0.002	Promotes B-cell survival and plays a role in the regulation of humoral immunity. Activates NF-kappa-B and JNK.
Cyp51	Cytochrome P450, family 51	0.78	p = 0.009	Electron carrier activity and heme binding
Sapcd1	Suppressor APC domain containing 1	1.22	p = 0.010	Unknown
Syt17	Synaptotagmin XVII	1.32	p = 0.006	Unknown
Igj	Immunoglobulin joining chain	0.21	p = 0.005	Immunoglobulin joining chain; humoral immune response (KEGG Pathway)
Ighg	Immunoglobulin heavy chain (gamma polypeptide)	0.19	p = 0.002	Immunoglobulin heavy chain(gamma polypeptide)
Ifi27l2b	Interferon, alpha-inducible protein 27 like 2B	0.76	p = 0.008	Unknown
Ddc	dopa decarboxylase	1.26	p = 0.009	Catecholamine biosynthesis
6330403K07Rik	RIKEN cDNA 6330403K07 gene	1.31	p = 0.007	Unknown
Slc35d3	Solute carrier family 35, member D3	1.29	p = 0.006	Carbohydrate transmembrane transport
Rhobtb1	Rho-related BTB domain containing 1	1.23	p = 0.009	Small GTPase mediated signal transduction
Myo5a	Myosin VA	0.72	p = 0.004	Actin filament-based movement
Hmgcs2	3-hydroxy-3-methylglutaryl-Coenzyme A synthase 2	1.23	p = 0.007	Cholesterol metabolic process; Synthesis and degradation of ketone bodies (KEGG Pathway)
Anln	Anillin, actin binding protein	1.29	p = 0.006	Cytokinesis
Agpat3	1-acylglycerol-3-phosphate O-acyltransferase 3	0.78	p = 0.007	Phospholipid biosynthesis
Ntf3	Neurotrophin 3	1.34	p = 0.008	Cell fate determination; MAPK signaling pathway (KEGG Pathway)
Chic1	Cysteine-rich hydrophobic domain 1	1.41	p = 0.007	Transport
Dusp1	Dual specificity phosphatase 1	1.43	p = 0.009	Protein amino acid dephosphorylation
Zkscan6	Zinc finger with KRAB and SCAN domains 6	0.79	p = 0.010	Unknown
Dclk1	Double cortin-like kinase 1	0.78	p = 0.006	Unknown
Kcnip4	Kv channel interacting protein 4	1.39	p = 0.007	Potassium ion transport
C030039L03Rik	RIKEN cDNA C030039L03 gene	1.23	p = 0.008	Unknown
Ide	Insulin degrading enzyme	0.71	p = 0.010	Negative regulation of proteolysis; Alzheimer’s disease (KEGG Pathway)
Rasgef1b	RasGEF domain family, member 1B	1.26	p = 0.006	Cytokinesis; cell proliferation; positive regulation of GTPase activity
Traf4	TNF receptor associated factor 4	0.78	p = 0.008	Respiratory gaseous exchange; respiratory tube development; programmed cell death
