# Corrigendum: Pathophysiological mechanisms underlying phenotypic differences in pulmonary radioresponse

**DOI:** 10.1038/srep46782

**Published:** 2017-05-04

**Authors:** Isabel L. Jackson, Yuji Zhang, Søren M. Bentzen, Jingping Hu, Angel Zhang, Zeljko Vujaskovic

Scientific Reports 6: Article number: 3657910.1038/srep36579; published online: 11 15 2016; updated: 05 04 2017

This Article contains errors. In the legend of Figure 1,

“1.25 cGy min^−1^”

should read:

“1.25 Gy min^−1^”.

In addition, in the Methods section under subheading ‘Animals and Radiation Exposure’,

“For serial tissue analysis, age and sex-matched C57BL/6J and C57L/J mice at 10–12 weeks of age were irradiated to the whole lung with a single dose of 15 Gy of 320 kVp X-rays at a dose rate of 1.25 cGy min^−1^”

should read:

“For serial tissue analysis, age and sex-matched C57BL/6J and C57L/J mice at 10–12 weeks of age were irradiated to the whole lung with a single dose of 15 Gy of 320 kVp X-rays at a dose rate of 1.25 Gy min^−1^”

