# Supplementary material for: Pathophysiological mechanisms underlying phenotypic differences in pulmonary radioresponse
Source: Sci Rep. 2016 Nov 15;6:36579. doi: 10.1038/srep36579 (PMC5109047; doi:10.1038/srep36579)
Supplement: Supplementary Information [file srep36579-s1.pdf]

Pathophysiological mechanisms underlying phenotypic differences in pulmonary radioresponse

Authors: Isabel L. Jackson[1,*,†], Yuji Zhang[2], Søren M. Bentzen[2], Jingping Hu[1], Angel Zhang[1], Zeljko Vujaskovic[1, †]

Affiliations:

[1]Division of Translational Radiation Sciences, Department of Radiation Oncology, University of Maryland School of Medicine, Baltimore, MD 21201.[2]

[2]Division of Biostatistics and Bioinformatics, Department of Epidemiology & Public Health, and the Greenebaum Cancer Center, University of Maryland School of Medicine, Baltimore, MD 21201

† Authors were previously affiliated with the Department of Radiation Oncology, Duke University Medical Center, Durham, NC 27710.

***Corresponding Author:**

Isabel L. Jackson, PhD

Division of Translational Radiation Sciences, Department of Radiation Oncology

685 W. Baltimore Street, Medical Sciences Teaching Facility, Room 7-00A

Baltimore, MD 21201

Phone: 410-706-5139

Fax: 410-706-2626

Email: ijackson@som.umaryland.edu

SUPPLEMENTAL MATERIAL

Figure 1. Clustered heat-map of genes (n = 805) significantly differentially expressed in response to pulmonary irradiation. Changes in gene expression is presented by murine strain and radiation dose. The intensity of gene expression is depicted from low (light blue) to high (dark blue).

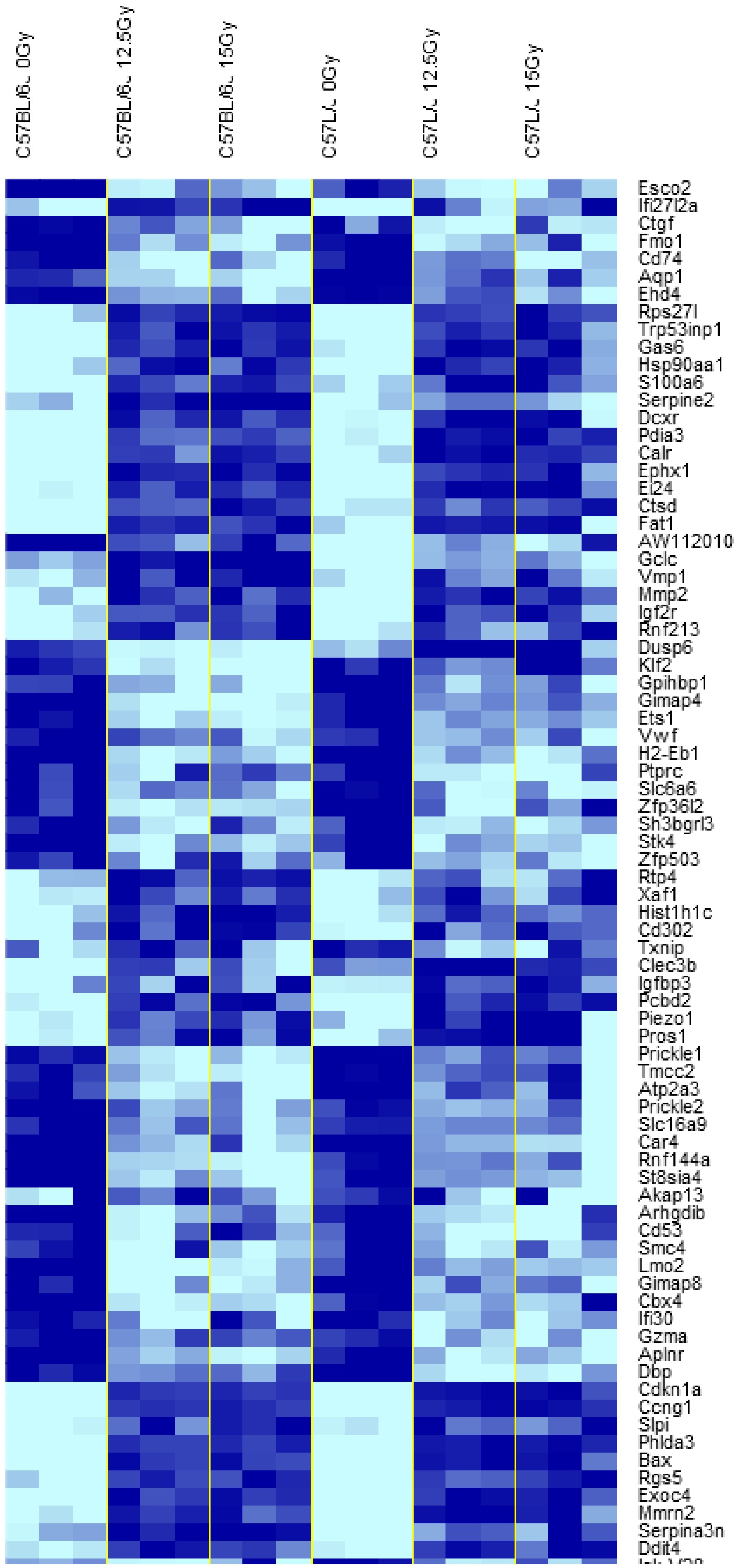
C57BL/6J 0Gy
C57BL/6J 12.5Gy
C57BL/6J 15Gy
C57L/J 0Gy
C57L/J 12.5Gy
C57L/J 15Gy
Esco2
Ifi27l2a
Ctgf
Fmo1
Cd74
Aqp1
Ehd4
Rps27l
Trp53inp1
Gas6
Hsp90aa1
S100a6
Serpine2
Dcxr
Pdia3
Calr
Ephx1
Ei24
Ctsd
Fat1
AW112010
Gclc
Vmp1
Mmp2
Igf2r
Rnf213
Dusp6
Klf2
Gpihbp1
Gimap4
Ets1
Vwf
H2-Eb1
Ptprc
Slc6a6
Zfp36l2
Sh3bgrl3
Stk4
Zfp503
Rtp4
Xaf1
Hist1h1c
Cd302
Txnip
Clec3b
Igfbp3
Pcbd2
Piezo1
Pros1
Prickle1
Tmcc2
Atp2a3
Prickle2
Slc16a9
Car4
Rnf144a
St8sia4
Akap13
Arhgdib
Cd53
Smc4
Lmo2
Gimap8
Cbx4
Ifi30
Gzma
Aplnr
Dbp
Cdkn1a
Ccng1
Slpi
Phlda3
Bax
Rgs5
Exoc4
Mmrn2
Serpina3n
Ddit4

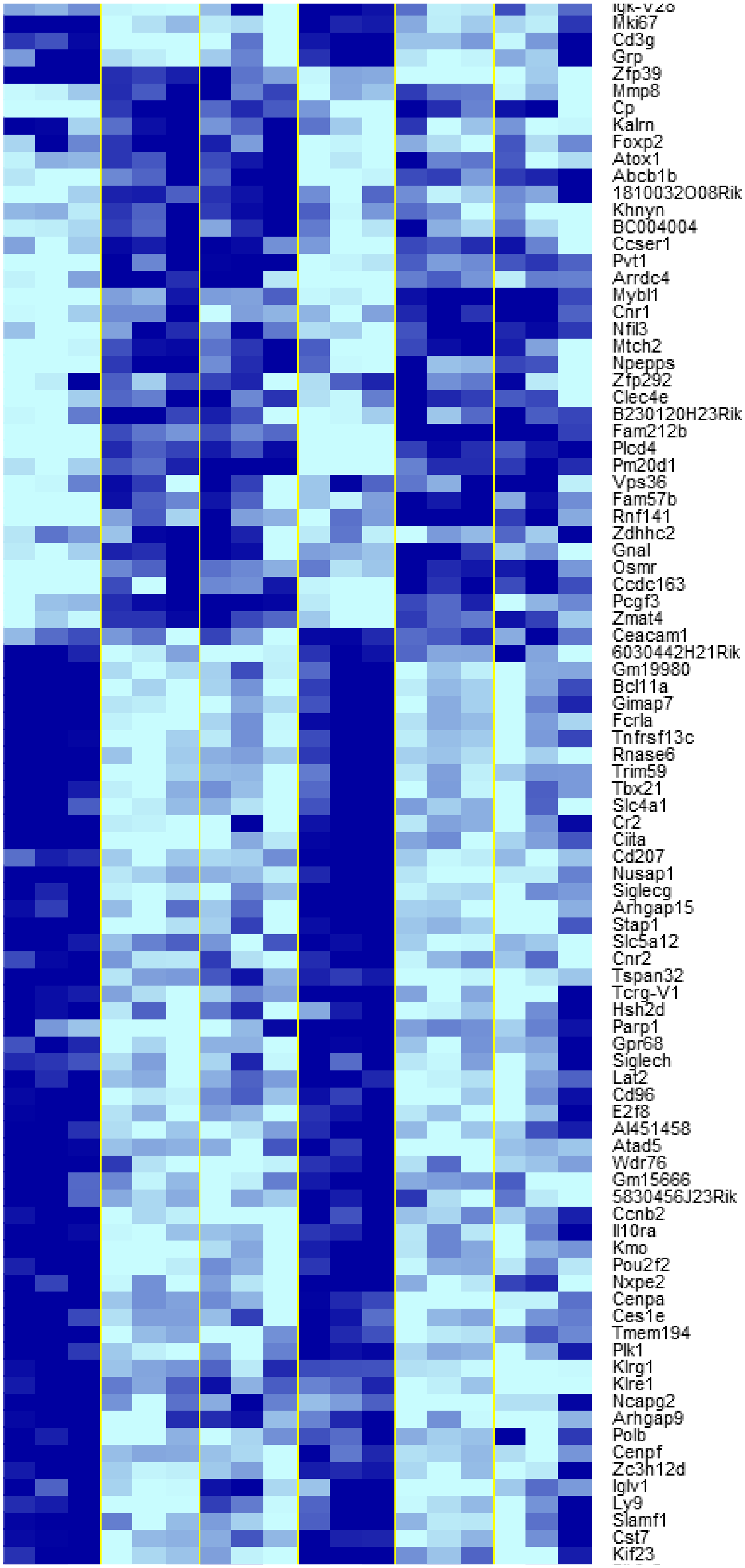
Mki67
Cd3g
Grp
Zfp39
Mmp8
Cp
Kalrn
Foxp2
Atox1
Abcb1b
1810032O08Rik
Khnyn
BC004004
Ccser1
Pvt1
Arrdc4
Mybl1
Cnr1
Nfil3
Mtch2
Npepps
Zfp292
Clec4e
B230120H23Rik
Fam212b
Plcd4
Pm20d1
Vps36
Fam57b
Rnf141
Zdhhc2
Gnal
Osmr
Ccdc163
Pcgf3
Zmat4
Ceacam1
6030442H21Rik
Gm19980
Bcl11a
Gimap7
Fcrla
Tnfrsf13c
Rnase6
Trim59
Tbx21
Slc4a1
Cr2
Ciita
Cd207
Nusap1
Siglecg
Arhgap15
Stap1
Slc5a12
Cnr2
Tspan32
Tcrg-V1
Hsh2d
Parp1
Gpr68
Siglech
Lat2
Cd96
E2f8
AI451458
Atad5
Wdr76
Gm15666
5830456J23Rik
Ccnb2
Il10ra
Kmo
Pou2f2
Nxpe2
Cenpa
Ces1e
Tmem194
Plk1
Klrg1
Klre1
Ncapg2
Arhgap9
Polb
Cenpf
Zc3h12d
Iglv1
Ly9
Slamf1
Cst7
Kif23

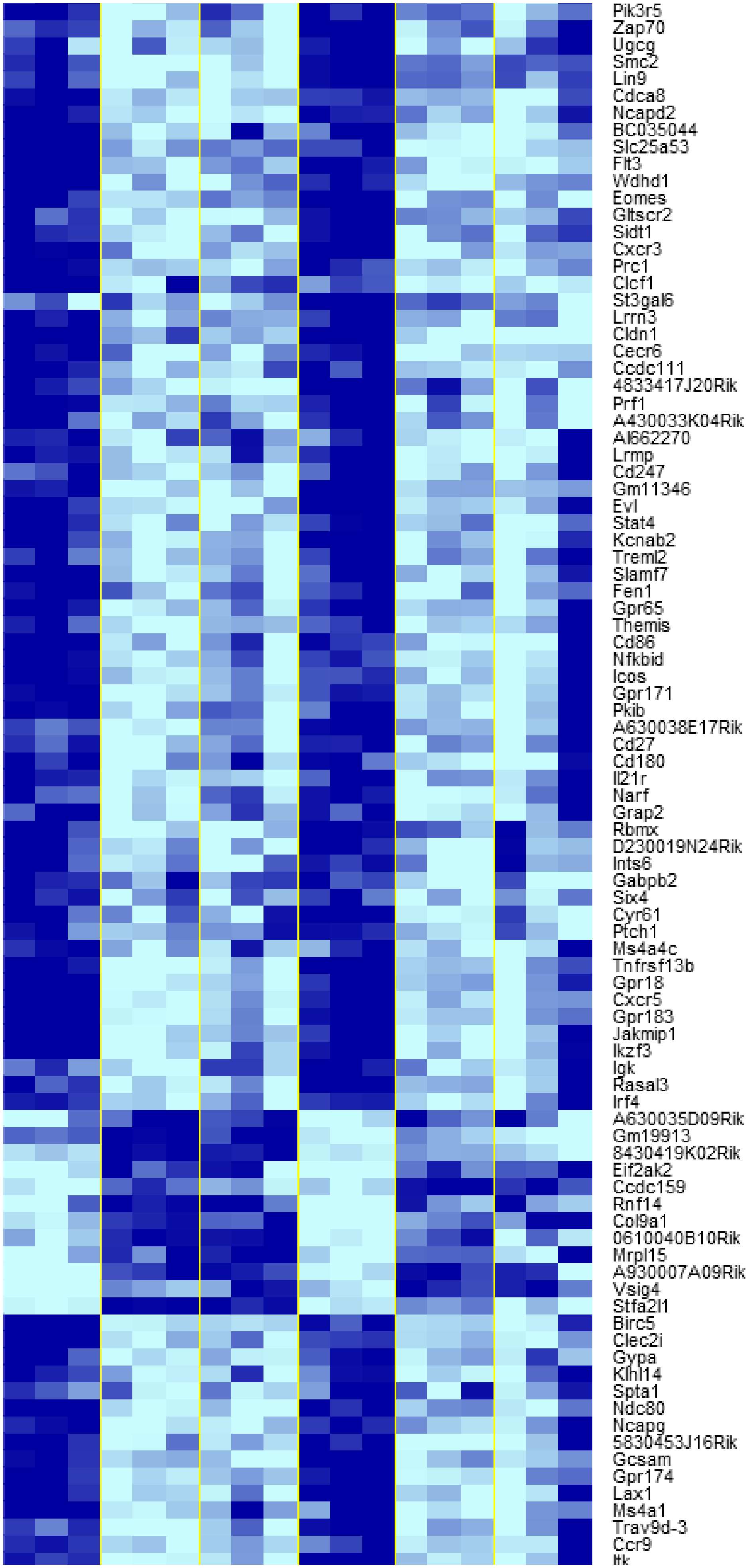

Pik3r5
Zap70
Ugcg
Smc2
Lin9
Cdca8
Ncapd2
BC035044
Slc25a53
Flt3
Wdhd1
Eomes
Gltscr2
Sidt1
Cxcr3
Prc1
Clcf1
St3gal6
Lrrn3
Cldn1
Cecr6
Ccdc111
4833417J20Rik
Prf1
A430033K04Rik
AI662270
Lrmp
Cd247
Gm11346
Evl
Stat4
Kcnab2
Treml2
Slamf7
Fen1
Gpr65
Themis
Cd86
Nfkbid
Icos
Gpr171
Pkib
A630038E17Rik
Cd27
Cd180
Il21r
Narf
Grap2
Rbmx
D230019N24Rik
Ints6
Gabpb2
Six4
Cyr61
Ptch1
Ms4a4c
Tnfrsf13b
Gpr18
Cxcr5
Gpr183
Jakmip1
Ikzf3
Igk
Rasal3
Irf4
A630035D09Rik
Gm19913
8430419K02Rik
Eif2ak2
Ccdc159
Rnf14
Col9a1
0610040B10Rik
Mrpl15
A930007A09Rik
Vsig4
Stfa2l1
Birc5
Clec2i
Gypa
Klhl14
Spta1
Ndc80
Ncapg
5830453J16Rik
Gcsam
Gpr174
Lax1
Ms4a1
Trav9d-3
Ccr9

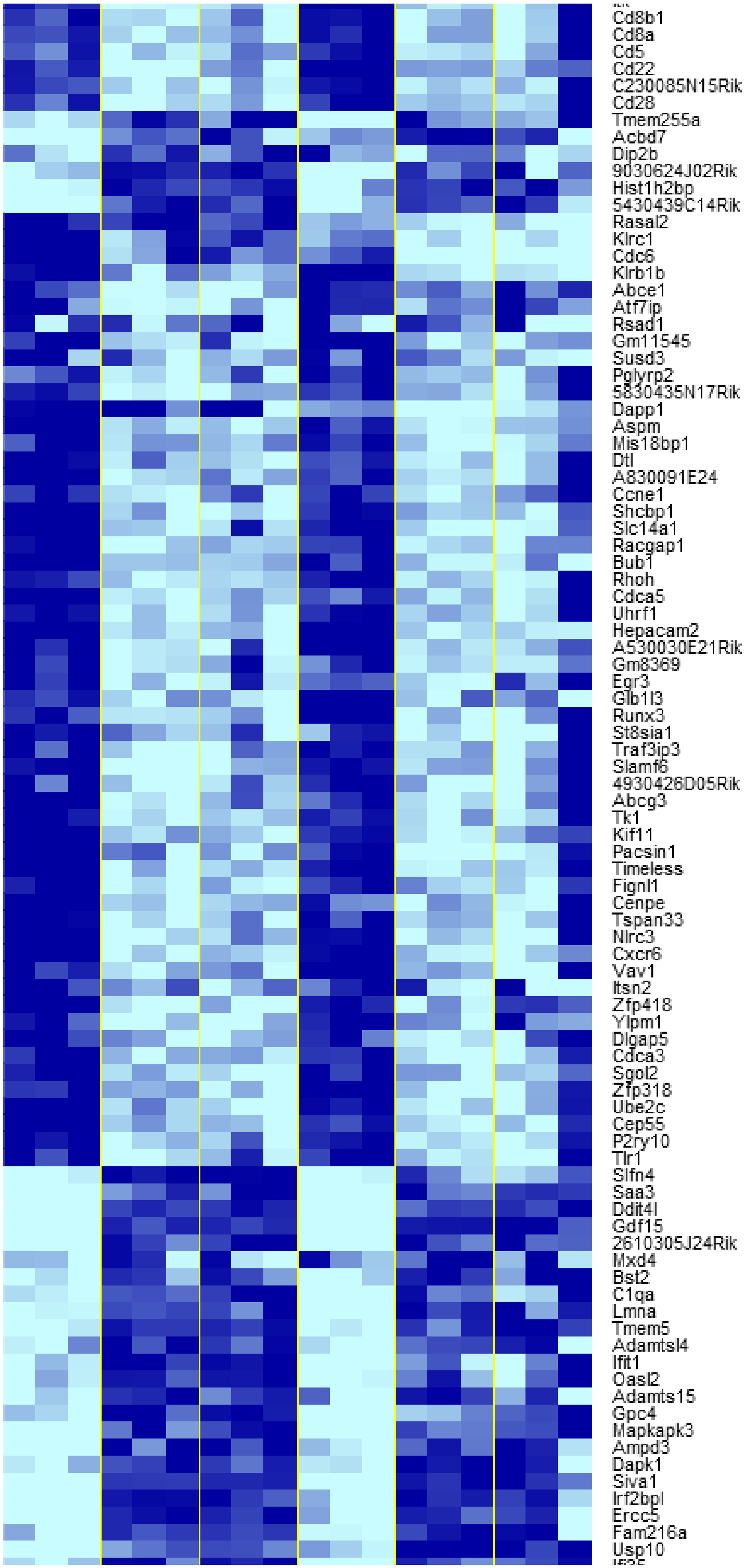

Cd8b1
Cd8a
Cd5
Cd22
C230085N15Rik
Cd28
Tmem255a
Acbd7
Dip2b
9030624J02Rik
Hist1h2bp
5430439C14Rik
Rasal2
Klrc1
Cdc6
Klrb1b
Abce1
Atf7ip
Rsad1
Gm11545
Susd3
Pglyrp2
5830435N17Rik
Dapp1
Aspm
Mis18bp1
Dtl
A830091E24
Ccne1
Shcbp1
Slc14a1
Racgap1
Bub1
Rhoh
Cdca5
Uhrf1
Hepacam2
A530030E21Rik
Gm8369
Egr3
Glb1l3
Runx3
St8sia1
Traf3ip3
Slamf6
4930426D05Rik
Abcg3
Tk1
Kif11
Pacsin1
Timeless
Fignl1
Cenpe
Tspan33
Nlrc3
Cxcr6
Vav1
Itsn2
Zfp418
Ylpm1
Dlgap5
Cdca3
Sgol2
Zfp318
Ube2c
Cep55
P2ry10
Tlr1
Slfn4
Saa3
Ddit4l
Gdf15
2610305J24Rik
Mxd4
Bst2
C1qa
Lmna
Tmem5
Adamtsl4
Ifit1
Oasl2
Adamts15
Gpc4
Mapkapk3
Ampd3
Dapk1
Siva1
Irf2bpl
Ercc5
Fam216a
Usp10

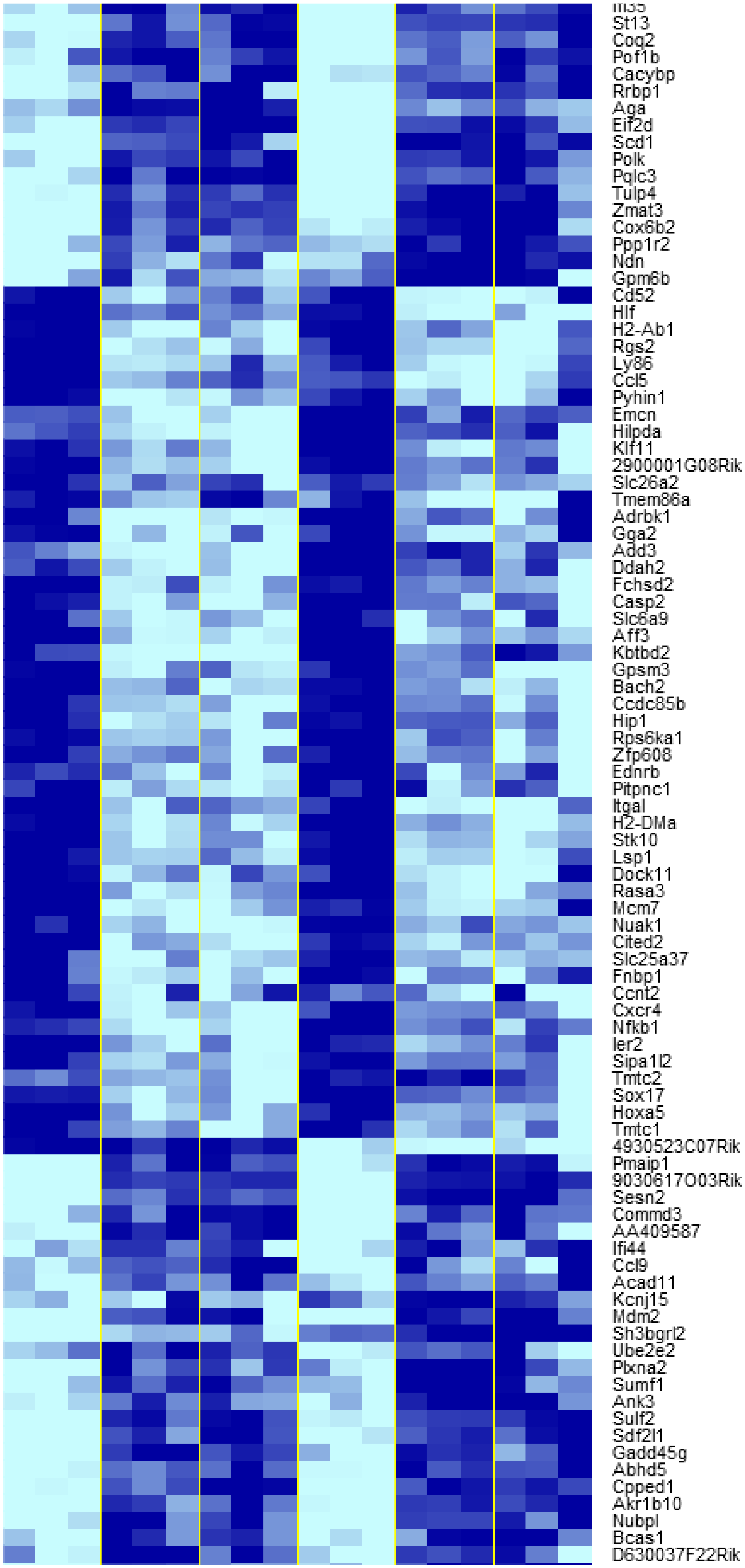
St13
Coq2
Pof1b
Cacybp
Rrbp1
Aga
Eif2d
Scd1
Polk
Pqlc3
Tulp4
Zmat3
Cox6b2
Ppp1r2
Ndn
Gpm6b
Cd52
Hlf
H2-Ab1
Rgs2
Ly86
Ccl5
Pyhin1
Emcn
Hilpda
Klf11
2900001G08Rik
Slc26a2
Tmem86a
Adrbk1
Gga2
Add3
Ddah2
Fchsd2
Casp2
Slc6a9
Aff3
Kbtbd2
Gpsm3
Bach2
Ccdc85b
Hip1
Rps6ka1
Zfp608
Ednrb
Pitpnc1
Itgal
H2-DMa
Stk10
Lsp1
Dock11
Rasa3
Mcm7
Nuak1
Cited2
Slc25a37
Fnbp1
Ccnt2
Cxcr4
Nfkb1
Ier2
Sipa1l2
Tmtc2
Sox17
Hoxa5
Tmtc1
4930523C07Rik
Pmaip1
9030617O03Rik
Sesn2
Commd3
AA409587
Ifi44
Ccl9
Acad11
Kcnj15
Mdm2
Sh3bgrl2
Ube2e2
Plxna2
Sumf1
Ank3
Sulf2
Sdf2l1
Gadd45g
Abhd5
Cpped1
Akr1b10
Nubpl
Bcas1
D630037F22Rik

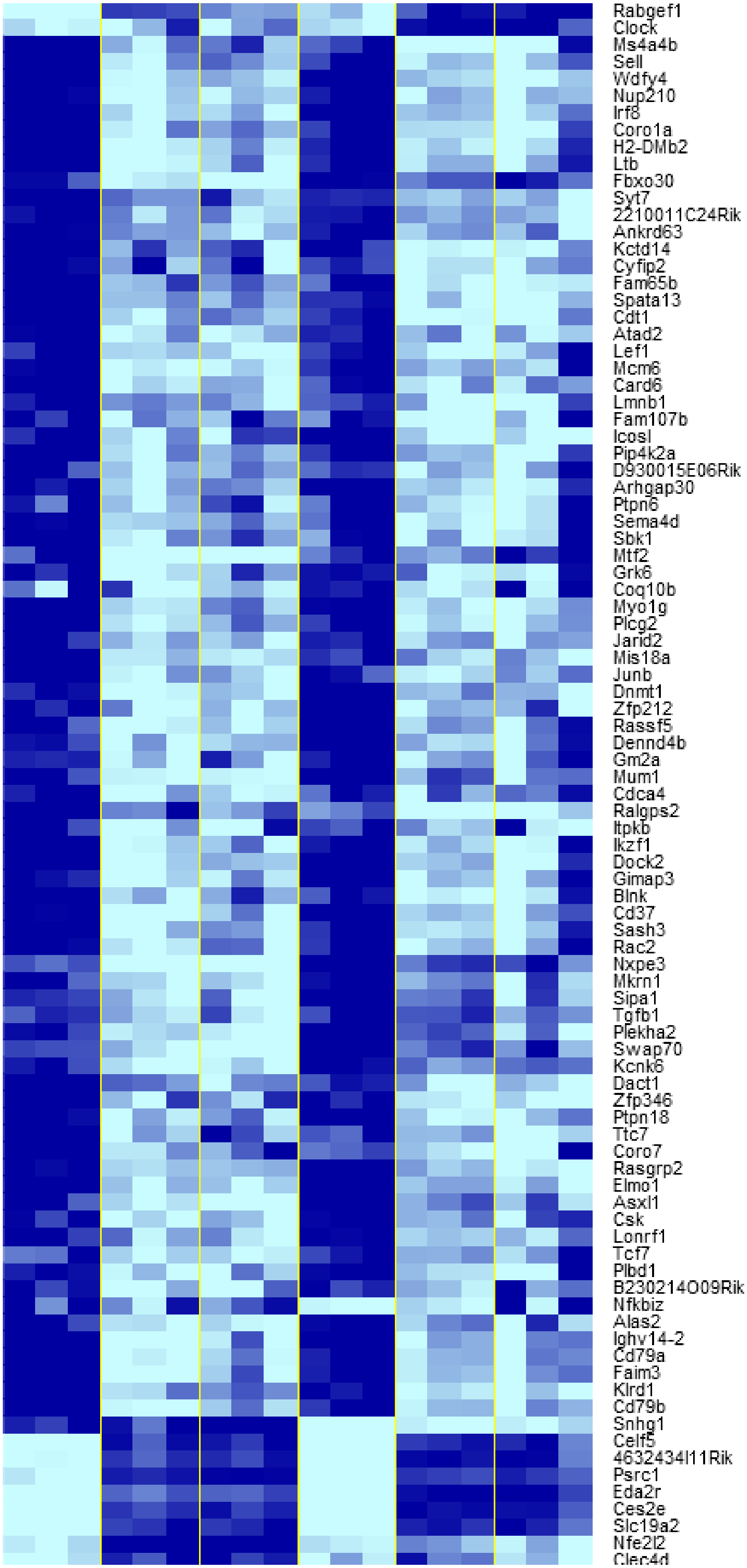
Rabgef1
Clock
Ms4a4b
Sell
Wdfy4
Nup210
Irf8
Coro1a
H2-DMb2
Ltb
Fbxo30
Syt7
2210011C24Rik
Ankrd63
Kctd14
Cyfip2
Fam65b
Spata13
Cdt1
Atad2
Lef1
Mcm6
Card6
Lmnb1
Fam107b
Icosl
Pip4k2a
D930015E06Rik
Arhgap30
Ptpn6
Sema4d
Sbk1
Mtf2
Grk6
Coq10b
Myo1g
Plcg2
Jarid2
Mis18a
Junb
Dnmt1
Zfp212
Rassf5
Dennd4b
Gm2a
Mum1
Cdca4
Ralgps2
Itpkb
Ikzf1
Dock2
Gimap3
Blnk
Cd37
Sash3
Rac2
Nxpe3
Mkrn1
Sipa1
Tgfb1
Plekha2
Swap70
Kcnk6
Dact1
Zfp346
Ptpn18
Ttc7
Coro7
Rasgrp2
Elmo1
Asxl1
Csk
Lonrf1
Tcf7
Plbd1
B230214O09Rik
Nfkbiz
Alas2
Ighv14-2
Cd79a
Faim3
Klrd1
Cd79b
Snhg1
Celf5
4632434I11Rik
Psrc1
Eda2r
Ces2e
Slc19a2
Nfe2l2
Clec4d

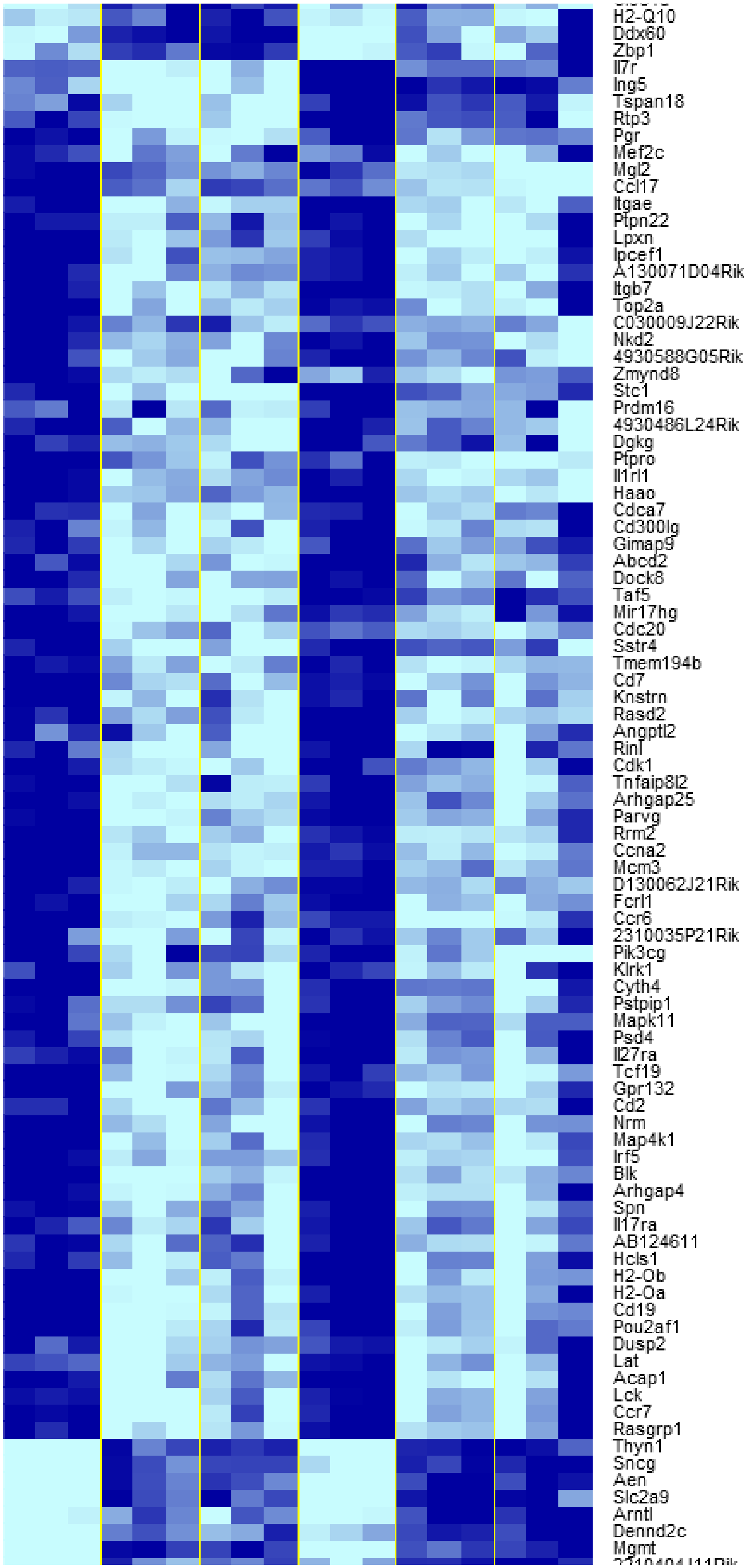
H2-Q10
Ddx60
Zbp1
Il7r
Ing5
Tspan18
Rtp3
Pgr
Mef2c
Mgl2
Ccl17
Itgae
Ptpn22
Lpxn
Ipcef1
A130071D04Rik
Itgb7
Top2a
C030009J22Rik
Nkd2
4930588G05Rik
Zmynd8
Stc1
Prdm16
4930486L24Rik
Dgkg
Ptpro
Il1rl1
Haao
Cdca7
Cd300lg
Gimap9
Abcd2
Dock8
Taf5
Mir17hg
Cdc20
Sstr4
Tmem194b
Cd7
Knstrn
Rasd2
Angptl2
Rinl
Cdk1
Tnfaip8l2
Arhgap25
Parvg
Rrm2
Ccna2
Mcm3
D130062J21Rik
Fcrl1
Ccr6
2310035P21Rik
Pik3cg
Klrk1
Cyth4
Pstpip1
Mapk11
Psd4
Il27ra
Tcf19
Gpr132
Cd2
Nrm
Map4k1
Irf5
Blk
Arhgap4
Spn
Il17ra
AB124611
Hcls1
H2-Ob
H2-Oa
Cd19
Pou2af1
Dusp2
Lat
Acap1
Lck
Ccr7
Rasgrp1
Thyn1
Sncg
Aen
Slc2a9
Arntl
Dennd2c
Mgmt

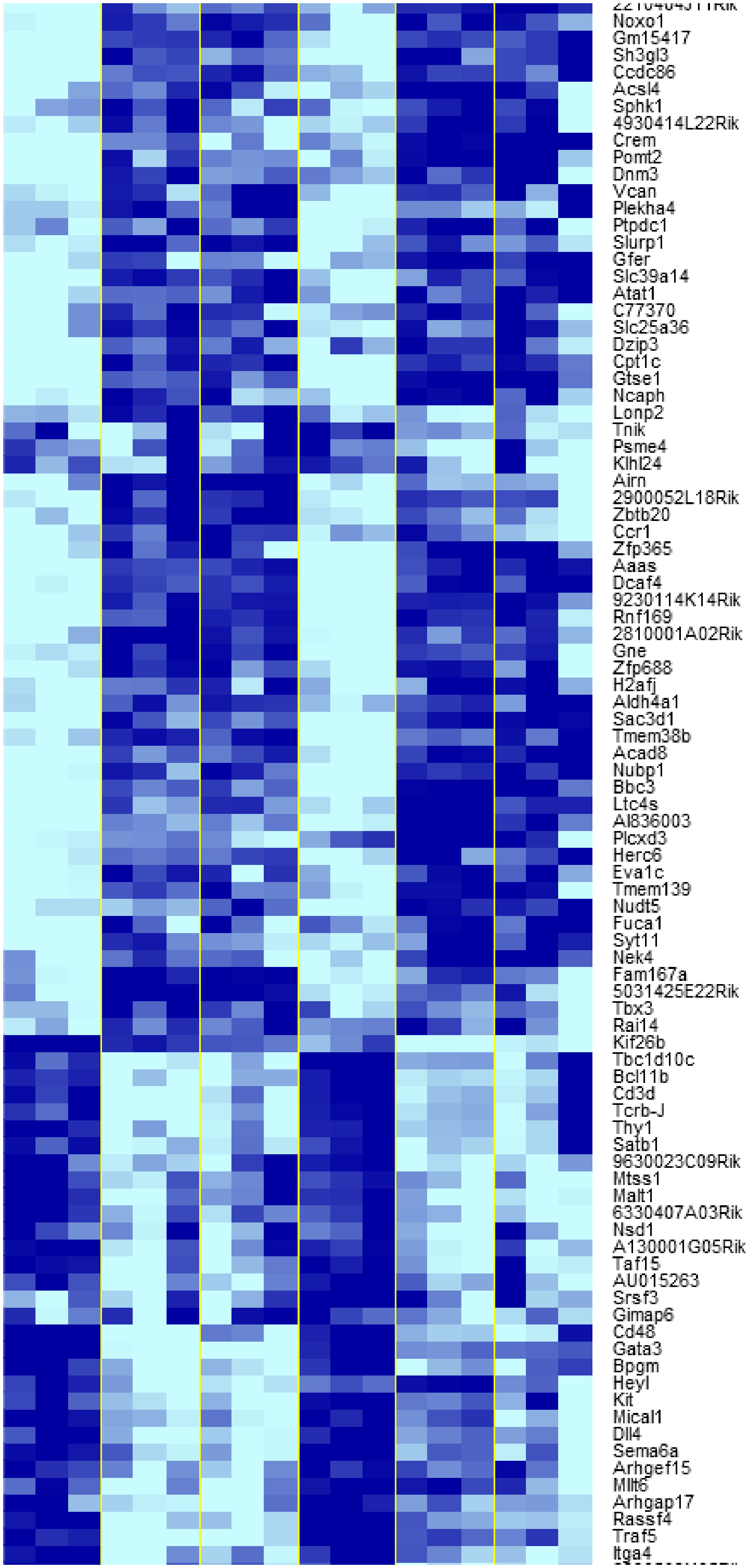
Noxo1
Gm15417
Sh3gl3
Ccdc86
Acsl4
Sphk1
4930414L22Rik
Crem
Pomt2
Dnm3
Vcan
Plekha4
Ptpdc1
Slurp1
Gfer
Slc39a14
Atat1
C77370
Slc25a36
Dzip3
Cpt1c
Gtse1
Ncaph
Lonp2
Tnik
Psme4
Klhl24
Airn
2900052L18Rik
Zbtb20
Ccr1
Zfp365
Aaas
Dcaf4
9230114K14Rik
Rnf169
2810001A02Rik
Gne
Zfp688
H2afj
Aldh4a1
Sac3d1
Tmem38b
Acad8
Nubp1
Bbc3
Ltc4s
AI836003
Plcxd3
Herc6
Eva1c
Tmem139
Nudt5
Fuca1
Syt11
Nek4
Fam167a
5031425E22Rik
Tbx3
Rai14
Kif26b
Tbc1d10c
Bcl11b
Cd3d
Tcrb-J
Thy1
Satb1
9630023C09Rik
Mtss1
Malt1
6330407A03Rik
Nsd1
A130001G05Rik
Taf15
AU015263
Srsf3
Gimap6
Cd48
Gata3
Bpgm
Heyl
Kit
Mical1
Dll4
Sema6a
Arhgef15
Mllt6
Arhgap17
Rassf4
Traf5
Itga4

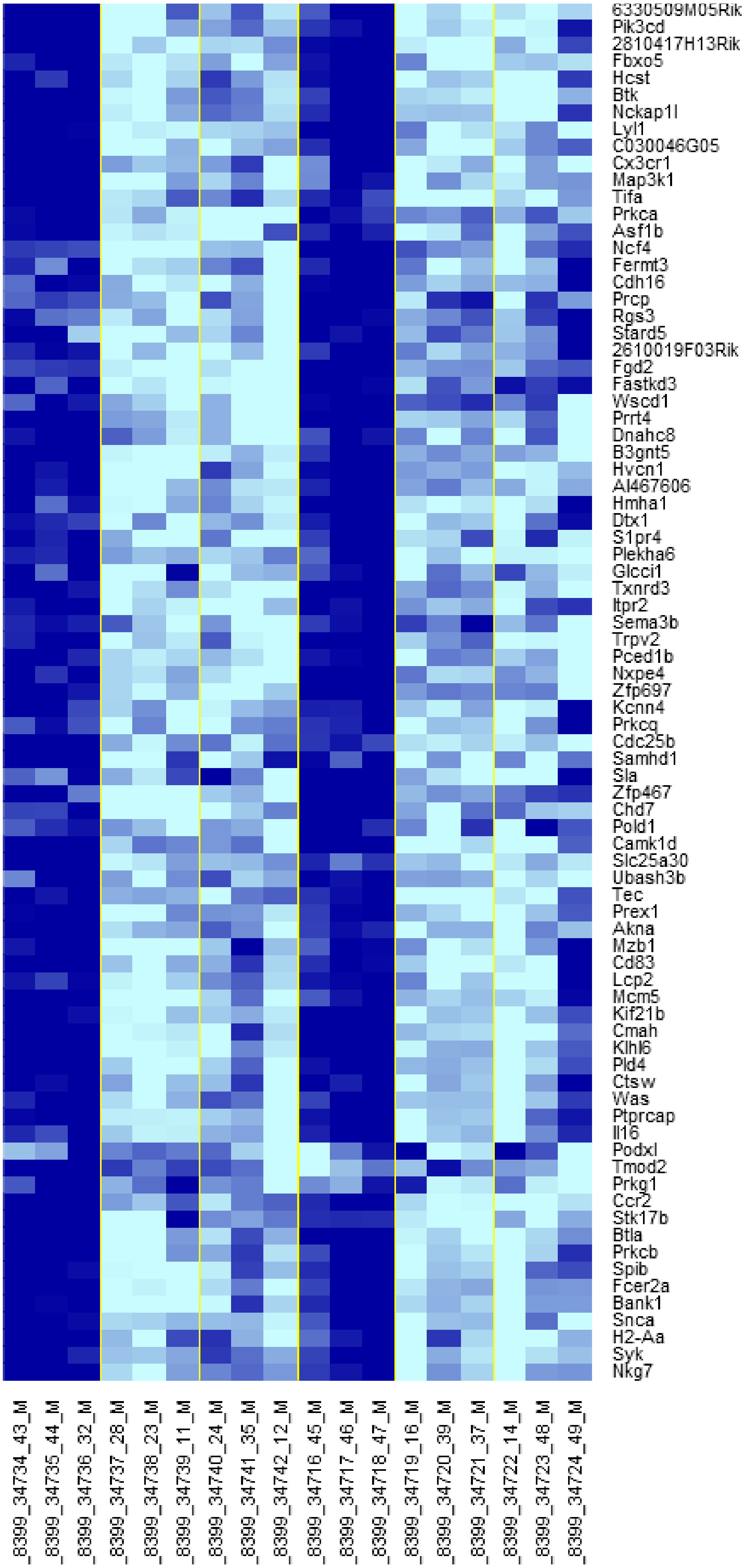
6330509M05Rik
Pik3cd
2810417H13Rik
Fbxo5
Hcst
Btk
Nckap1l
Lyl1
C030046G05
Cx3cr1
Map3k1
Tifa
Prkca
Asf1b
Ncf4
Fermt3
Cdh16
Prcp
Rgs3
Stard5
2610019F03Rik
Fgd2
Fastkd3
Wscd1
Prrt4
Dnahc8
B3gnt5
Hvcn1
AI467606
Hmha1
Dtx1
S1pr4
Plekha6
Glcci1
Txnrd3
Itpr2
Sema3b
Trpv2
Pced1b
Nxpe4
Zfp697
Kcnn4
Prkcq
Cdc25b
Samhd1
Sla
Zfp467
Chd7
Pold1
Camk1d
Slc25a30
Ubash3b
Tec
Prex1
Akna
Mzb1
Cd83
Lcp2
Mcm5
Kif21b
Cmah
Klhl6
Pld4
Ctsw
Was
Ptprcap
Il16
Podxl
Tmod2
Prkg1
Ccr2
Stk17b
Btla
Prkcb
Spib
Fcer2a
Bank1
Snca
H2-Aa
Syk
Nkg7
8399_34734_43_M
8399_34735_44_M
8399_34736_32_M
8399_34737_28_M
8399_34738_23_M
8399_34739_11_M
8399_34740_24_M
8399_34741_35_M
8399_34742_12_M
8399_34716_45_M
8399_34717_46_M
8399_34718_47_M
8399_34719_16_M
8399_34720_39_M
8399_34721_37_M
8399_34722_14_M
8399_34723_48_M
8399_34724_49_M

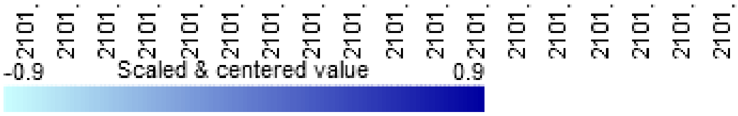

Data range before thresholding: -3.3 to 3.1.
Missing values are in color "gray".
